# Serum complement C3 and α_2_-macroglobulin are potentially useful biomarkers for inflammatory bowel disease patients

**DOI:** 10.1016/j.heliyon.2021.e06554

**Published:** 2021-03-23

**Authors:** Kohki Okada, Hiroshi Itoh, Masaki Ikemoto

**Affiliations:** a Department of Medical Technology and Sciences, Faculty of Health Sciences, Kyoto Tachibana University, Kyoto 607-8175, Japan; b Faculty of Bioscience, Nagahama Institute of Bio-Science and Technology, Shiga 526-0829, Japan

**Keywords:** Complement C3, α_2_-macroglobulin, Inflammatory bowel disease, Biomarker, Inflammatory cytokines, Clinical chemistry

## Abstract

**Aims:**

Ulcerative colitis (UC), characterized by chronic inflammation and its recurrence in the large intestine, is well known as inflammatory bowel disease (IBD). Suitable biomarkers specific for UC are poorly understood till date. We aimed to discover novel serum biomarkers for UC and identify good indicators that reflected the severity of UC.

**Main methods:**

Serum samples were obtained from out-patients with IBD (n = 101) and healthy volunteers (HVs, n = 101). Serum proteins were subjected to high performance liquid chromatography (HPLC) and sodium dodecyl sulfate-electrophoresis (SDS-PAGE) analysis. After electrophoresis, proteins in the gel were identified by mass spectrometry. Further, the protein concentration was measured by enzyme-linked immunosorbent assays (ELISAs). Based on the results, correlations between the serum levels of these proteins and the disease activity index scores for UC were statistically evaluated.

**Principal findings:**

HPLC showed that chromatograms of serum proteins from HVs apparently differed from those of patients with IBD. Eleven protein bands, which were different in their protein concentrations from those in HVs, were separated by SDS-PAGE accordingly. Among them, complement C3 (c-C3) and α_2_-macroglobulin (α_2_-MG), with high protein scores, were identified by mass spectrometry. The serum concentration of c-C3 in patients with IBD was higher than that in HVs. However, the level of α_2_-MG in patients with IBD was significantly lower than that in HVs. Hence, the serum levels of c-C3 and α_2_-MG could be good indicators of the severity of UC.

**Conclusion:**

Serum c-C3 and α_2_-MG are suitable biomarkers for monitoring the condition of patients with UC.

## 1 Introduction

Inflammatory bowel disease (IBD) is characterized by chronic inflammation and its recurrence in the large intestine. Ulcerative colitis (UC) and Crohn's disease (CD) are widely known as IBD by many clinicians. In most cases, the patients suffer from chronic abdominal pain with diarrhea, reiterate relapse, and remission of the disease (Sairenji et al., 2017). Although the cause of IBD is still unclear, there is a possibility that a hereditary predisposition in humans, such as NOD2/CARD15, IRGM, and ATG16L1, may be relevant to the onset of IBD (Watanabe et al., 2004; Massery and Parkes, 2007; Henckaerts et al., 2008). The lack of such genetic elements may result in abnormal immunoreaction to intestinal bacteria or other substances, thus leading to an inflammation in the intestinal tract. In addition, immune cells from myeloid origin, such as neutrophils and macrophages, may be deeply involved in the onset of IBD and its deterioration. Upon activation of immune cells in colonic tissues, plenty of inflammatory cytokines are secreted in circulation. As a result, these inflammatory cytokines unnecessarily attack epithelial cells. This leads to the destruction of the immunological barrier system in the body. To ameliorate abnormal macrophage functions, an immunosuppressive agent, e.g., tacrolimus (FK506), is currently used to treat IBD. It is clinically effective to maintain remission of IBD (Yoshino et al., 2010; Kvedaraite et al., 2016). However, accurate diagnosis of IBD by doctors is not easy owing to the complexity of the pathological condition in patients with IBD. Therefore, a suitable course of treatment is strongly desired in patients with IBD.

A colonoscopy is widely used to directly observe the inner condition of the large intestinal tract of patients with IBD. However, frequent colonoscopy is indeed hard for patients who are affected physically and psychologically with IBD. In addition, this method is not only time consuming in the hospital, but it is also expensive for the patients. For a long time, C-reactive protein (CRP) has been used as a useful biomarker for many inflammatory diseases all over the world (Pepys and Hirschfield, 2003). Alternatively, high-sensitive CRP (hsCRP) is also a useful clinical biomarker for chronic inflammation. However, both CRP and hsCRP are not necessarily specific for IBD (Mańkowska-Wierzbicka et al., 2016). Reportedly, fecal S100A8/A9 (calprotectin) is a more suitable biomarker for severe IBD, especially UC (Sipponen and Kolho, 2015; Puolanne et al., 2016; Patel et al., 2017). In addition, enzyme-linked immunosorbent assay (ELISA) for rat S100A9 (rS100A9) showed that the protein markedly increased in the stool of rats with experimental colitis induced with dextran sulfate sodium (DSS) (Sekiya et al., 2016). Our previous findings showed that rS100A9 are highly expressed in tissue macrophages in the large intestine of rats and are largely secreted from the cells (Okada et al., 2015). Additionally, S100A9 is deeply involved in the induction of intestinal inflammation and its subsequent aggravation in the large intestine. Furthermore, we previously showed that the increased level of rS100A8/A9 in the stool could reflect histological severity of experimental colitis in rats, and that the serum level of S100A8/A9 in patients with UC is a useful indicator to precisely evaluate the disease activity (Okada et al., 2019, 2020). We previously reported that rS100A8 and rS100A9 bind to CD68 on macrophages to regulate their immune functions in a coordinated manner. Presumably, lack of coordination between rS100A8 and rS100A9 in macrophages may be responsible for further deterioration in the state of patients with IBD (Okada et al., 2016). To date, we have paid attention to interleukin (IL)-6 and tumor necrosis factor (TNF)-α in patients with IBD; however, they are not always specific biomarkers for IBD (Cioffi et al., 2015; Norouzinia et al., 2017). Besides, many biomarkers for IBD, such as antiglycan antibodies, leucine-rich alpha-2 glycoprotein (LRG), and pyruvate kinase M2 (PKM2), in serum have been explored recently, but they had some weak points in sensitivity and specificity for diagnosis of IBD (Almousa et al., 2018; Shinzaki et al., 2017; Simondi et al., 2008). In comparison with other indicators, more suitable biomarkers, which can evaluate the severity of IBD, should be newly discovered in the serum of patients with IBD.

In this study, we aimed to discover useful biomarkers for precisely evaluating the severity of IBD using high performance liquid chromatography (HPLC), sodium dodecyl sulfate polyacrylamide gel electrophoresis (SDS-PAGE), and matrix assisted laser desorption/ionization time-of-flight mass spectrometry (MALDI-TOF MS) techniques.

## 2 Materials and methods

### 2.1 Ethics statement

Informed consent was obtained from all patients who participated in this study. In addition, all experiments were performed in conformity with the WMA Declaration of Helsinki-Ethical Principles for Medical Research Involving Human Subjects (64th WMA General Assembly; Fortaleza, Brazil, October 2013) and were approved by the ethics committee of Tenri Health Care University and Tenri Hospital (permission number: 069).

### 2.2 Blood samples

Human blood samples were obtained from out-patients with IBD in Tenri hospital, together with their medical data. At the same time, normal blood samples were obtained from healthy volunteers (HVs) diagnosed with no any diseases in Tenri hospital. All blood samples were centrifuged after coagulation. The resultant serum samples were transferred into polycarbonate tubes (1.5 mL) at an adequate volume and kept frozen at -80 °C until use. The concentrations of CRP and total proteins (TP) were measured by LABOSPECT 008 (HITACHI High Technologies corp., Tokyo, Japan). The number of platelets and white blood cells (WBC) was counted by XN-1000 (Sysmex corp., Hyogo, Japan). Patients have been individually prescribed with therapeutic agents, such as 5-aminosalicylic acid (5-ASA), immunomodulator, and prednisolone, since before they participate in this study; however, no treatment of anti-TNF therapy, leukocytapheresis, or any surgery had been received. The medical information (sex and age) relevant to each patient and the number of participants in this study is summarized in Table 1. Two gastroenterologists assessed the severity of UC by disease activity index (DAI) scores that ranged from 0 to 3. The criteria of DAI scores based on Truelove-Witts index are summarized in Table 2 (Truelove and Witts, 1954). All samples were subjected to the same procedures as the experiments of our previous study (Okada et al., 2019).

**Table 1:** Medical information of participants in this study.

Participants	Subjects		
	HVs	UC	CD
Number	101	61	40
Male	76	50	28
Female	25	11	12
Age (mean ± SD)	32.8 ± 13.4	55.9 ± 11.6	34.2 ± 8.6
Range	23–65	20–67	20–69
Median	25.0	57.5	37.4
Disease duration (months; mean ± SD)		82.4 ± 20.2	69.3 ± 24.5
Therapeutic agents			
5-ASA		61	40
Immunomodulator		38	22
Prednisolone		5	4
DAI score			
0		23	
1		34	
2		4	
3		0	

HVs, healthy volunteers; UC, ulcerative colitis; CD, Crohn's disease.

5-ASA, 5-aminosalicylic acid; DAI score, disease activity index score.

SD, standard deviation.

**Table 2:** Criteria of disease activity index score.

	Remission (score: 0)	Mild (score: 1)	Moderate (score: 2)	Severe (score: 3)
Stool frequency (per day)	1~2	≦ 4	Middle condition of severe and mild	≧ 6
Occult/Gross bleeding	(-)	(-)~(+)		(+++)
Fever	(-)	(-)		≧ 37.5 °C
Tachycardia	(-)	(-)		≧ 90/min
Anemia	(-)	(-)		≦ Hb 10 g/dL
ESR	Normal	Normal		≧ 30 mm/h

Hb, hemoglobin concentration in circulating blood.

ESR, erythrocyte sedimentation rate.

### 2.3 Reagents

ELISA kits for human inflammatory biomarkers, such as α_2_-macrogulobulin (α_2_-MG), IL-6, TNF-α, and IL-1β, were obtained from COSMO BIO Co. Ltd. (Tokyo, Japan). An ELISA kit for human complement C3 (c-C3) was purchased from Assaypro, LLC (Missouri, USA). All other reagents were obtained from Wakenyaku Co. Ltd. (Kyoto, Japan).

### 2.4 Protocol for HPLC

Human serum samples were analyzed by Agilent 1220 Infinity LC equipped with a gel filtration column (TSKgel G3000 SW, 30 cm × 7.8 mm, particle size: 5 μm, pore size: 250 A) (Tosoh Co. Ltd., Tokyo, Japan). All samples for HPLC analysis were confirmed to have similar values of TP (the mean ± standard deviation value was 73.4 ± 3.8 g/L) and were preliminarily diluted twice with 50 mM phosphate buffer solution/0.9% NaCl, pH 7.4. The column temperature was set at 25 °C, and the flow rate was kept at 1.0 ml/min using the same buffer. After injection of each sample (100 μl), proteins eluted from the column were detected at 280 nm. Each sample was analyzed for 20 min (as retention time) after the injection. Samples were collected from 6.33 to 10 min at an interval of 0.33 min/fraction.

### 2.5 SDS-PAGE

After HPLC, proteins in each fraction were further separated by SDS-PAGE in the presence of 2-mercaptoethanol as described previously (Towbin et al., 1979). The concentration of all polyacrylamide gels was 12.5%. Finally, coomassie brilliant blue (CBB) staining was carried out to visualize proteins bands in each gel.

### 2.6 MALDI-TOF mass spectrometry

After SDS-PAGE, some protein bands were cut out and delivered to MALDI-TOF Mass Spectrometry accession service in COSMO BIO Co. Ltd. Briefly, a single protein band was digested by trypsin and Peptide Mass Fingerprint (PMF) analysis was carried out with Microflex LRF 20 (Bruker Daltonics Corp., Massachusetts, USA). Fragments of protein components were identified by Mascot search on National Center for Biotechnology Information (NCBI) database. Mascot scores of >67 were regarded as significant.

### 2.7 ELISAs

ELISAs for human inflammatory biomarkers, such as c-C3, α_2_-MG, IL-6, TNF-α, and IL-1β, were performed according to the manufacturer's instructions. The reaction was stopped by adding 1N sulfuric acid, and the color reaction was measured at 490 nm with a microplate reader (Bio-Rad Laboratories, Inc., California, USA; iMark™).

### 2.8 Statistical analysis

Pair-wise comparisons with the controls were performed using parametric tests. Significant differences between groups were identified using Student's t-test. Data are shown as mean ± standard deviation values. Correlation between the two groups in each case was assessed by the Spearman test using the statistical software ‘Easy R’ (Kanda, 2013). The correlation coefficient (R-value) from 0.5 to 1.0 indicated good correlation. P-values of <0.05 were considered significant.

## 3 Results

### 3.1 Fractionation of serum samples by HPLC

**Figure 1:**
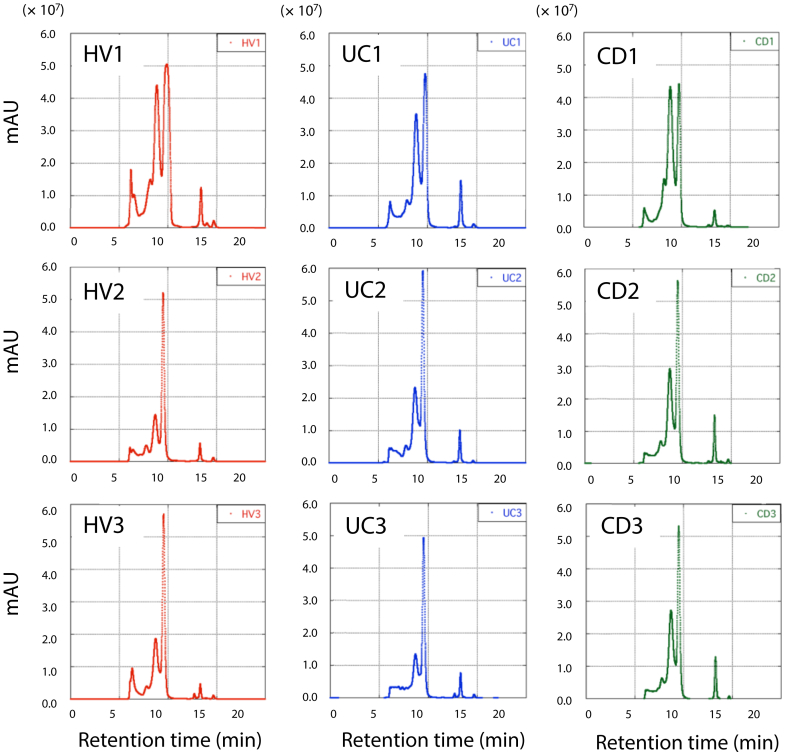
Fractionation of serum proteins in each sample by HPLC using a gel filtration column. Panels (HV1 to HV3, UC1 to UC3, and CD1 to CD3) indicate chromatograms of serum proteins from healthy volunteers (red), patients with UC (blue), and patients with CD (green), respectively. X-axis indicates retention time after the start. Y-axis indicates the absorbance at 280 nm. The HPLC protocol is described in the “*Materials and Methods”* section in detail.

**Figure 2:**
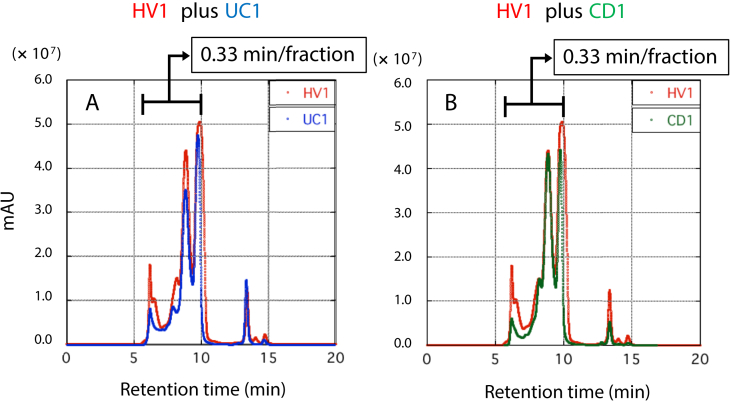
Comparison of the chromatograms of HV1 and UC1 or CD1 in an overlay manner. In A, the chromatogram of HV1 (red) was overlaid with that of UC1 (blue). In B, the chromatogram of HV1 (red) was overlaid with that of CD1 (green). X-axis indicates retention time after the start. Y-axis indicates the absorbance at 280 nm.

Serum proteins of the HVs and patients with IBD were fractionated by HPLC (n = 3 in each subject). Samples obtained from patients with UC or CD with three highest values of CRP in each group were subjected to HPLC analysis. The chromatograms of the HVs were different from those of patients with IBD in their elution patterns (Figure 1). Especially, the samples of HV1 and patients with UC1 or CD1 seemed to have various kinds of proteins compared with those of other subjects. To show the difference more clearly, chromatograms of the HV1 were overlaid on those of patients with UC1 or CD1 (Figure 2). We found that the protein content of the fractions distinctly differed in each sample during the retention time from 6.33 to 10 min. Hence, eleven fractions were separately collected at the interval of 0.33 min during this time period.

### 3.2 Separation and selection of serum proteins in each fraction by SDS-PAGE

**Figure 3:**
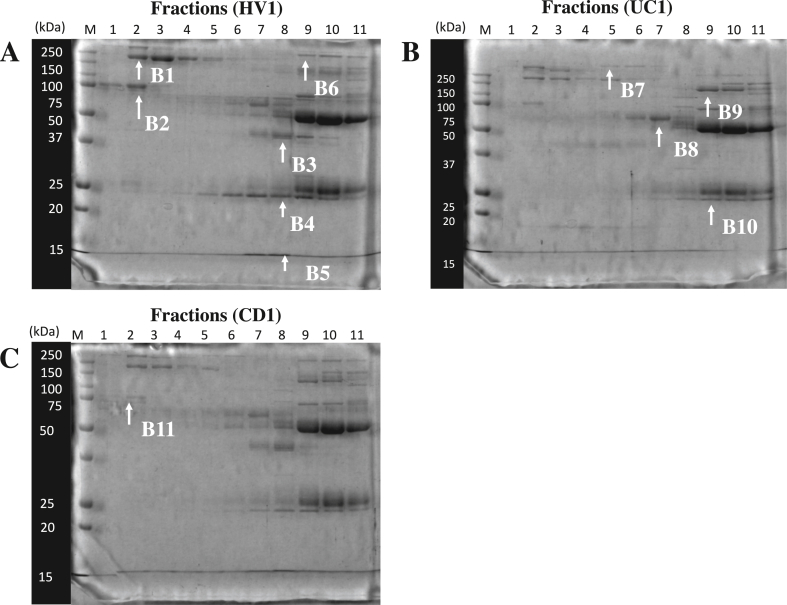
Separation of serum proteins in each fraction by SDS-PAGE. SDS-PAGE was carried out in the presence of 2-mercaptoethanol (2-ME) using serum proteins in each fraction, which was obtained by HPLC. A, B, and C show protein bands in eleven fractions from HV1, UC1, and CD1 serum, respectively. Lane M indicates molecular weight markers. Lanes 1 to 11 indicate eleven fractions after HPLC. Protein bands of B1 to B11 were subjected to mass spectrometry (MALDI-TOF).

SDS-PAGE was further carried out to separate serum proteins in each fraction after HPLC. We carefully looked at each protein band in the gel and selected eleven protein bands (B1–B11) with different densities from HV1 and patients with UC1 or CD1 (Figure 3). These protein bands were subjected to mass spectrometry.

### 3.3 Identification of serum proteins by mass spectrometry

To identify eleven protein bands in the gel after SDS-PAGE, each band was separately cut out from the gels. As analyzed by mass spectrometry (MALDI-TOF, COSMO BIO Co. Ltd., Japan), except for a protein band (B6), all the residual ten protein bands provided good protein scores. Consequently, 16 proteins (P1-1 to P11) were identified based on the information available from the NCBI database. The results were summarized in Table 3. After detailed consideration, we paid attention to alpha-2-macroglobulin isoform X1 (P1-1) and complement C3 preproprotein (P9) with high mascot scores (the bold fonts in Table 3) and predicted that serum α_2_-MG and c-C3 will have potential utility as useful biomarkers for IBD.

**Table 3:** Identification of protein components via NCBI database.

Band	Sample	Results analyzed	Score
B1	**P1-1**	**alpha-2-macroglobulin isoform X1**	**204**
	P1-2	unnamed protein product	87
B2	P2	Keratin 1	126
B3	P3-1	Keratin 1	87
	P3-2	Keratin type1cytoskeletal 9	78
	P3-3	Haptoglobin Precursor protein	73
B4	P4-1	Proapolipoprotein, partial	151
	P4-2	Immunoglobulin light chain, partial	69
B5	P5-1	Mutant beta-globin	109
	P5-2	Hemoglobin alpha-1 globin chain	101
B7	P7-1	Inter-alpha-trypsin inhibitor heavy chain H2 precursor	102
	P7-2	Inter-alpha-trypsin inhibitor heavy chain ITIH1	72
B8	P8	Unnamed protein product	70
B9	**P9**	**Complement C3 preproprotein**	**197**
B10	P10	Immunoglobulin variable region, partial	78
B11	P11	Full Ig mu chain C region	122

### 3.4 Quantitative measurement of the serum c-C3 and α_2_-MG in HVs and patients with IBD by ELISAs

**Figure 4:**
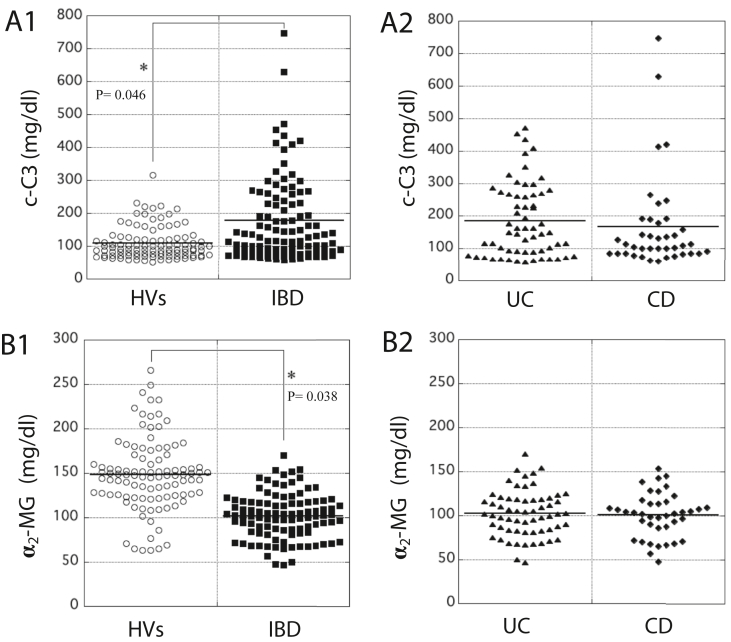
Measurement of the concentration of c-C3 and α_2_-MG in the serum of HVs and patients with IBD by ELISAs. In A1 and B1, the concentration of c-C3 (mg/dl) and α_2_-MG (mg/dl), respectively, in the serum of HVs and patients with IBD was measured using each ELISA kit for c-C3 or α_2_-MG. Panels A2 and B2 show that data obtained from patients with IBD were further classified into two groups of patients, UC and CD. Average in each case is indicated by a horizontal bar. The color reaction was measured at 490 nm with an iMark microplate reader (BIO-RAD, Richmond, CA).

As determined by ELISAs, the serum concentration (177 mg/dl) of c-C3 in the patients with IBD was significantly higher than that (109 mg/dl) in HVs. The value (average) in both groups is indicated by a horizontal bar (Figure 4, panel A1). In terms of fluctuation of serum c-C3 concentration, almost no significant difference between patients with UC and CD was observed (Figure 4, panel A2). In contrast, the serum concentration (102 mg/dl) of α_2_-MG in the patients with IBD was significantly lower than that (149 mg/dl) in HVs. The value (average) in both groups is indicated by a horizontal bar (Figure 4, panel B1). Almost no significant difference in the distribution of serum α_2_-MG concentration was observed between the patients with UC and CD (Figure 4, panel B2).

### 3.5 Correlation between the concentrations of c-C3 and CRP or inflammatory cytokines in patients with IBD

**Figure 5:**
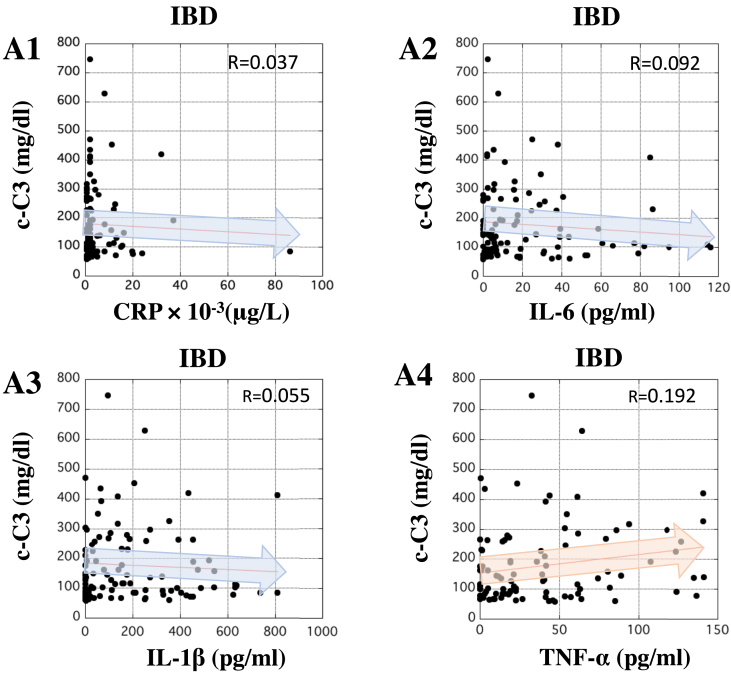
Correlation between the concentrations of c-C3 and inflammatory biomarkers in the serum of patients with IBD. In panels A1 to A4, X-axis indicates the concentration of CRP (μg/L), IL-6, IL-1β, and TNF-α (pg/ml), respectively, in the serum of patients with IBD. In all panels, Y-axis indicates the concentration of c-C3 (mg/dl) in the serum of patients with IBD. The correlations between c-C3 and the inflammatory biomarkers in patients with IBD were analyzed by Pearson analysis using ‘Easy R’ software (Kanda, 2013). R-value between 0.5 and 1.0 was considered statistically significant.

The correlation between serum concentrations of c-C3 and inflammatory biomarkers in patients with IBD was examined. The serum concentration of c-C3 barely correlated with that of CRP in patients with IBD. Additionally, the concentration of c-C3 did not correlate with that of IL-6, IL-1β, or TNF-α in the patients (Figure 5, panels A1 to A4). These results suggested that serum c-C3 might have clinical significance over other inflammatory biomarkers.

### 3.6 Correlation between the serum concentrations of α_2_-MG and CRP or inflammatory cytokines in patients with IBD

**Figure 6:**
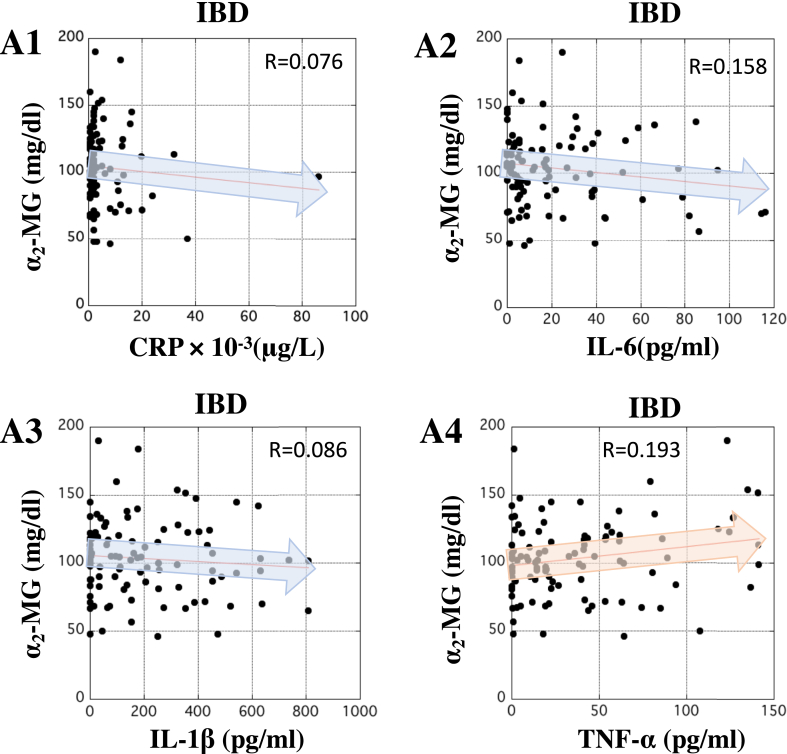
Correlation between the concentration of α_2_-MG and inflammatory biomarkers in the serum of patients with IBD. In panels A1 to A4, X-axis indicates the concentration of CRP (μg/L), IL-6, IL-1β, and TNF-α (pg/ml), respectively, in the serum of patients with IBD. In all panels, Y-axis indicates the concentration of α_2_-MG (mg/dl) in the serum of patients with IBD. The correlations between α_2_-MG and the inflammatory biomarkers in patients with IBD were analyzed by Pearson analysis using ‘Easy R’ software (Kanda, 2013). R-value between 0.5 and 1.0 was considered statistically significant.

The correlation between the concentrations of α_2_-MG and inflammatory markers was also investigated. In terms of serum level, slight correlation between α_2_-MG and CRP, IL-6, IL-1β, or TNF-α in patients with IBD was observed (Figure 6, panels A1 to A4). These data suggest that serum α_2_-MG also provides another clinical advantage for monitoring the condition of patients with IBD.

### 3.7 Correlation between the serum concentration of c-C3 or α_2_-MG and the number of platelets or WBC in patients with IBD

**Figure 7:**
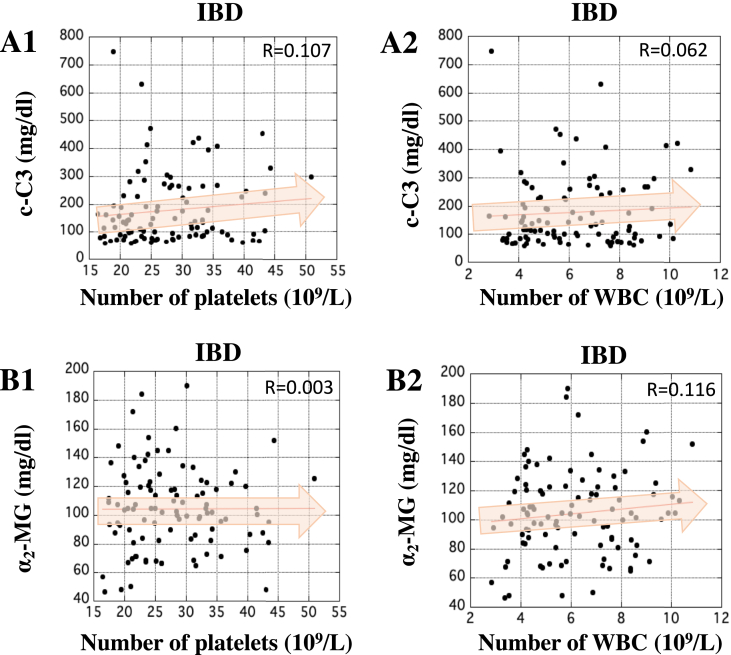
Correlation between the serum concentration of c-C3 or α_2_-MG and the number of platelets or WBC in patients with IBD. In A1 and A2, X-axis indicates the number of platelets and WBC, respectively, and Y-axis indicates the serum concentration of c-C3 (mg/dl). In B1 and B2, X-axis indicates the numbers of platelets and WBC, respectively, and Y-axis indicates the serum concentration of α_2_-MG (mg/dl). The correlations between the serum concentration of c-C3 or α_2_-MG and the number of platelets or WBC in patients with IBD were analyzed by Pearson analysis using ‘Easy R’ software (Kanda, 2013). R-value between 0.5 and 1.0 was considered statistically significant.

We examined the relationship between the serum concentration of c-C3 or α_2_-MG and the number of platelets or WBC using the whole blood obtained from patients with IBD. No correlation between the serum concentration of c-C3 and the number of platelets or WBC was observed (Figure 7, panels A1 and A2). Similarly, the serum concentration of α_2_-MG didn't correlate to the number of platelets or WBC (Figure 7, panels B1 and B2). These data suggest that the serum concentration of c-C3 or α_2_-MG does not share clinical significance of the number of platelets or WBC.

### 3.8 Correlation between DAI score and the serum concentration of c-C3 or α_2_-MG in patients with UC

**Figure 8:**
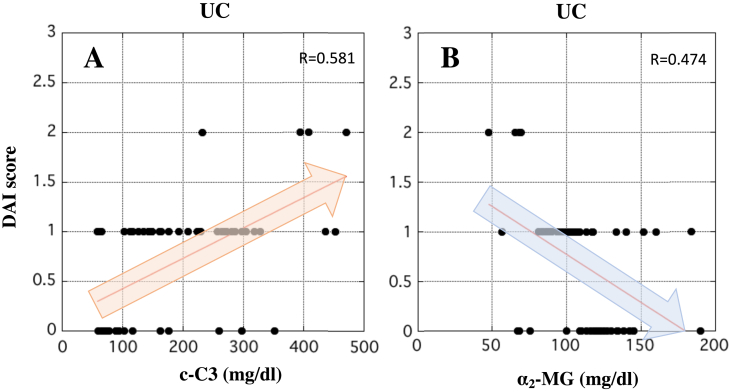
Correlation between the DAI score and the concentration of c-C3 or α_2_-MG in the serum of patients with UC. The severity of UC was indicated by the DAI score as described previously. In panels A and B, X-axis indicates the concentration of c-C3 (mg/dl) and α_2_-MG (mg/dl), respectively, in the serum of patients with UC. Y-axis indicates the DAI score. The correlation between the DAI score and the concentration of c-C3 or α_2_-MG in the serum of patients with UC was analyzed by Pearson analysis using ‘Easy R’ software (Kanda, 2013). R-value between 0.5 and 1.0 was considered statistically significant.

The correlation between the DAI score and the serum concentration of c-C3 or α_2_-MG in patients with UC was evaluated statistically. In patients with UC, the DAI score was directly proportional to the concentration of c-C3 as shown by R-value (0.581) (Figure 8A). Conversely, the DAI score was inversely proportional to the serum concentration of α_2_-MG (R-value = 0.474) (Figure 8B). In any case, these results suggest potential utility of c-C3 and α_2_-MG as biomarkers for reflecting the severity of UC.

## 4 Discussion

In this study, we described new biomarkers to correctly understand the disease state of patients with IBD and presented data supporting their clinical application. Among serum proteins identified by mass spectrometry, c-C3 and α_2_-MG were noteworthy as suitable biomarkers for IBD. The correlations between the serum concentration of c-C3 or α_2_-MG and other inflammatory biomarkers are helpful for evaluating their clinical significance. Our aim was to verify whether both c-C3 and α_2_-MG are different from other inflammatory biomarkers generally used in clinical significance and whether the two proteins could be useful indexes for the severity of UC.

We previously reported that serum S100A8/A9 was a potentially sensitive biomarker for IBD (Okada et al., 2019). Despite a significant increase in serum S100A8/A9 in the patients with IBD compared to HVs, there was almost no significant correlation between the DAI score and the serum concentration of S100A8/A9 (R = 0.342). Although the reason for this discrepancy is not clear, it seems that the serum concentration of S100A8/A9 in patients with IBD may slightly increase in the blood after the incidence of the disease. Based on the hypothesis that more useful biomarkers exist in the serum of patients with IBD, the serum components in the participants were fractionated by a HPLC system equipped with a gel filtration column using the serum samples, with the highest values of CRP, obtained from patients with UC or CD in each group (n = 3 each). As a result, the chromatograms of patients with UC1 and CD1 were different in their fraction patterns in comparison with those of HV1. Also, a significant difference in the chromatograms was observed even between UC1 and CD1. This observation suggests that some valuable biomarkers in circulation could exist in the patients with IBD (Figure 1). As shown by the HPLC chromatograms, eleven protein bands were apparently different in their concentrations, which was clearly observed. These proteins were then subjected to mass spectrometry to identify several kinds of proteins in the sample. The proteins identified were accordingly summarized in Table 3.

Among them, alpha-2-macroglobulin isoform X1 and complement C3 preproprotein, which have high mascot score, were candidates for new biomarkers for IBD. However, we found that the measurement of c-C3 preproprotein or α_2_-MG isoform X1 in hospitals was very difficult, even if it is possible. Here, we temporarily hypothesized that the analogous proteins, c-C3 and α_2_-MG, may be useful biomarkers for IBD. Based on our hypothesis, we tried to measure the serum concentrations of these biomarkers by ELISAs. As determined by ELISAs, the serum concentration of c-C3 in patients with IBD was significantly higher than that in HVs. However, a difference in the serum concentration of c-C3 between the patients with UC and CD was not observed although the two diseases belong to the IBD group (Figure 4, panels A1 and A2). This observation strongly suggests that c-C3 could be a useful biomarker for IBD. Alternatively, the serum concentration of α_2_-MG in patients with IBD was significantly lower than that in HVs (Figure 4, panel B1). Similar to c-C3, no significant difference in the serum concentration of α_2_-MG was seen among the patients with UC and CD (Figure 4, panel B2). Taken together, the change in the serum level of c-C3 was inversely proportional to that of α_2_-MG in patients with IBD. Nevertheless, these two proteins are suggestive biomarkers for IBD. As shown by lower R-values, no significant correlation between c-C3 and CRP, IL-6, IL-1β, or TNF-α was also observed, which is clearly visible (Figure 5). Also, α_2_-MG was not significantly correlated with such inflammatory biomarkers (Figure 6). Although the increase in the number of platelets or WBC in the blood is generally an important signal for acute inflammatory responses in the body, the changes in serum concentrations of c-C3 and α_2_-MG in patients with IBD were not correlated with those in these parameters (Figure 7). These results might afford an advantage to c-C3 and α_2_-MG over inflammatory biomarkers available. We assume that serum c-C3 and α_2_-MG may correctly reflect the status of chronic inflammation, but not acute, in IBD. Next, the disease severity was classified by DAI score to clinically evaluate the condition of patients with UC. Based on the DAI scores, the correlation between the severity of UC and the concentration of c-C3 or α_2_-MG was statistically analyzed; the concentration of serum c-C3 significantly correlated with the DAI score (R = 0.581), while that of α_2_-MG also showed a good inverse correlation coefficient (R = 0.474) (Figure 8). Our previous study showed the correlation between DAI score and serum S100A8/A9, CRP, or inflammatory cytokines in patients with UC; however, all R-values (<0.35) were lower than those of serum c-C3 and α_2_-MG (Okada et al., 2019). This fact is suggestive of the superiority of serum c-C3 and α_2_-MG to other laboratory biomarkers for IBD.

Many investigators have reported that c-C3 and α_2_-MG are not always specific for IBD, but rather the former could reflect the activity of other inflammatory diseases, such as vasculitis, glomerulosclerosis, and rheumatoid arthritis (Cosio and Hernandez, 1996; Crnogorac et al., 2018; Romano et al., 2018). Currently, there is a need for more reliable biomarkers for IBD as sought by many clinicians. For such requirements, c-C3 and α_2_-MG may be clinically applicable for monitoring the condition of patients with IBD before and after the onset of disease.

c-C3 is an important factor for complement activation in classical, alternative, and lectin pathways (Alcorlo et al., 2015). Because the complement system contributes to stimulation of phagocytes, activation of production of antibodies, and secretion of some cytokines by several triggers, c-C3 may be deeply involved in the regulation of autoimmune diseases including IBD (Nesargikar et al., 2012). Considering the immunological variation in diseases, c-C3 in the serum of patients with IBD should be a helpful indicator for directly understanding the severity of IBD. In contrast, the relationship between α_2_-MG and IBD may be interesting from the viewpoint of the self-regulation system in autoimmune diseases. Generally, α_2_-MG acts as a protease inhibitor and negatively regulates coagulation by suppressing activation of thrombin (de Boer et al., 1993). α_2_-MG reportedly behaves as a carrier protein that binds with some cytokines and/or growth factors to harmonize the immune system (Gonias et al., 2000). The specific mobility of α_2_-MG for many autoimmune diseases is suggestive of its clinical usefulness as a limited biomarker for these diseases. However, we have not found many reports that clearly describe the relation between α_2_-MG and IBD. Thus, this study is noteworthy as no correlation exists between α_2_-MG and other inflammatory biomarkers. This may lead to the discovery of a better biomarker to reflect the severity of IBD in the field of clinical laboratory medicine. Lately, some reports appeared that serum LRG and PKM2 would be useful indicators for judging the severity of IBD (Almousa et al., 2018; Shinzaki et al., 2017). However, a few methods to measure the both proteins were developed recently, but they are not widely spread in general hospitals yet. In that respect, c-C3 and α_2_-MG are popular proteins, so the two proteins are easily subject to ELISA, turbidimetric immunoassay, and nephelometry (Zhang et al., 2003; Nezu et al., 2013). Then, serum c-C3 and α_2_-MG in patients with IBD are more available than the other markers, such as LRG and PKM2, in clinical laboratory medicine.

In conclusion, we found that both c-C3 and α_2_-MG are useful biomarkers for IBD, especially for the severity of UC, and that the two proteins are superior to other biomarkers of IBD. The superiority of c-C3 and α_2-_MG to other inflammatory proteins, however, is not fully explained yet. Therefore, to explain the mechanism for fluctuation in their circulation, a study is currently in progress using rats with DSS-induced experimental colitis.

## Declarations

### Author contribution statement

Kohki Okada: Conceived and designed the experiments; Performed the experiments; Analyzed and interpreted the data; Contributed reagents, materials, analysis tools or data; Wrote the paper.

Hiroshi Itoh and Masaki Ikemoto: Conceived and designed the experiments; Analyzed and interpreted the data; Wrote the paper.

### Funding statement

This work was supported by 10.13039/501100001691JSPS KAKENHI for Young Scientists (JP 17K15786) and Japanese Society of Laboratory Medicine Fund for the Promotion of Scientific Research.

### Data availability statement

Data included in article/supplementary material/referenced in article.

### Declaration of interests statement

The authors declare no conflict of interest.

### Additional information

No additional information is available for this paper.
